# The Pararectus approach: a preferred surgical approach for fixation of acetabular fractures predominantly involving the anterior column – a narrative review

**DOI:** 10.1007/s00402-024-05455-7

**Published:** 2024-07-30

**Authors:** Thomas Freude, Dietmar Krappinger, Richard A. Lindtner, Fabian Stuby

**Affiliations:** 1 Department of Orthopaedics and Traumatology, Paracelsus Medical University Salzburg, Salzburg, Austria; 2 Department of Orthopaedics and Traumatology, Medical University of Innsbruck, Innsbruck, Austria; 3 Department of Traumatology and General Surgery, BG Unfallklinik Murnau, Murnau Am Staffelsee, Germany

**Keywords:** Acetabulum, Acetabular fractures, Ilioinguinal approach, Modified Stoppa approach, Pararectus approach, Anterior column, Posterior column, Anterior column posterior hemitransverse fracture, Surgical approach

## Abstract

Beginning in France in the 1960s, the management of acetabular fractures has increasingly evolved toward surgical treatment strategies. The basic principles established by the pioneers of acetabular surgery, Letournel and Judet - anatomical reconstruction of the joint and stable osteosynthesis - remain unchanged. Modern advancements in surgical techniques aim to reduce access-related trauma and minimize complications. The notable rise in acetabular fractures among the elderly, which predominantly affect the anterior aspects of the acetabulum, has driven the development of less invasive, soft tissue-sparing anterior approaches. This evolution began with the ilio-inguinal approach in the 1960s, progressed to the modified Stoppa approach in the 2000s and, most recently, the Pararectus approach in the 2010s. Each of these approaches upholds the fundamental principles of effective acetabular fracture care, while offering distinct advantages and disadvantages. In this review, we examine the merits and limitations of the Pararectus approach, specifically focusing on its utility in the surgical treatment of anterior column posterior hemitransverse acetabular fractures. Ultimately, the success of the individual patient’s outcome is less dependent on the chosen approach and more on the surgeon’s experience and expertise. Ideally, surgeons should be proficient in all these approaches to tailor the surgical strategy to the individual patient’s requirements, thereby ensuring optimal outcomes.

## Introduction

About 5 decades ago, the prevailing conservative treatment of acetabular fractures was revolutionized by Judet and Letournel [1; 2]. Their outstanding work was based on extensive studies of the micro- and macro-anatomy of the pelvis and the diverse fracture patterns of the acetabulum. They subsequently translated this knowledge into clinical practice, developing a classification and treatment algorithm for acetabular fractures that remain valid to this day.

In the 1960s, most acetabular fractures were caused by high energy traumas, such as car or two-wheeler accidents. These fractures exhibited a specific pathology location and primarily involved the posterior aspect of the acetabulum (posterior wall and posterior column) [3] or represented T-shaped fractures [4]. The current gold standard for the treatment of the posterior aspect of the acetabulum is the Kocher-Langenbeck approach [5].

In countries with a higher life expectancy and higher levels of activity among individuals aged 60 and older, an increase in the total number of acetabular fractures has been observed over the past two decades. Most notably, this increase was primarily attributed to the significant increase in older patients. Letournel and Judet have postulated that surgeons should not operate on patients over the age of sixty who have suffered an acetabular fracture, due to lower chances of surgical success. However, this surgical law of the fathers of pelvic surgery is no longer tenable [2; 5; 6]. The percentage distribution of fracture types has also changed to the same extent, with a decrease in acetabular fractures affecting the posterior structures (i.e. posterior wall, posterior column) and a significant increase in fracture types affecting the anterior aspect of the acetabulum or both columns [7].

**Fig. 1:**
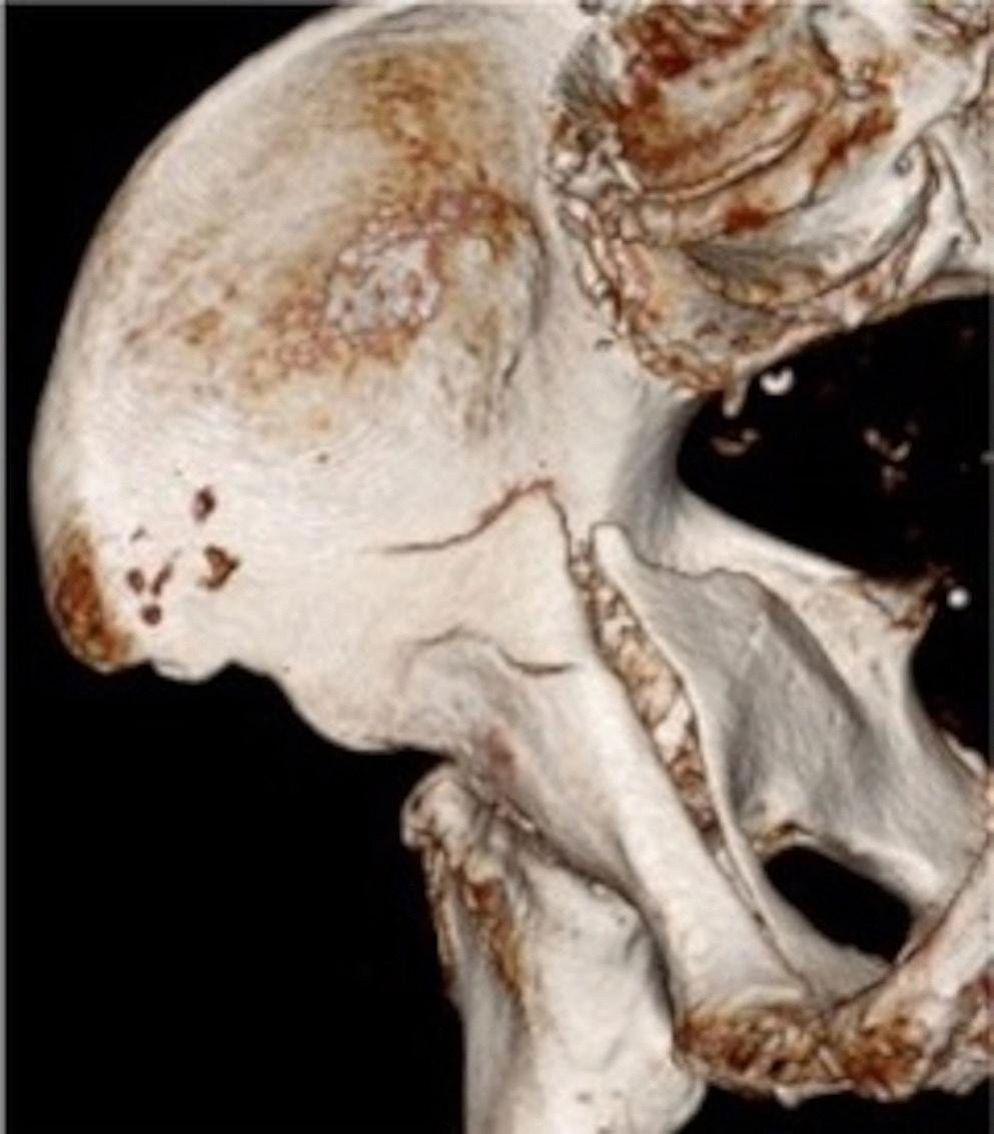
3-D CT reconstruction of an anterior column posterior hemitransverse acetabular fracture (ACPH)

These fracture types are typically caused by a lateral fall on the hip, with direct transmitted forces from the lateral parts of the proximal femur over the femoral neck and the femoral head to the acetabulum. The typical resulting fracture patterns are anterior column, anterior column posterior hemitransverse (ACPH) and two-column fractures [8]. The ACPH fracture is the most common type of fracture in older active individuals and is also the type of fracture that particularly qualifies for the Pararectus approach. A precise analysis of the anterior component of the ACPH fracture pattern typically shows a combination of a complex, multi-fragmentary fracture of the anterior column, medial protrusion of the femoral head and impaction of the superomedial articular surface (Gull Sign) [7; 9–11]. The posterior component of the ACPH pattern exhibits a simple posterior hemitransverse fracture and, distal to this a fracture, a internally rotated lower posterior column and ischium connected to the quadrilateral plate, which is ventrally swiveled open (referred to as a ‘door leaf)’ (Fig. 1) [12–14].

For many years, the ilioinguinal (or also called Letournel) approach has been the established surgical approach for the anterior aspects of the acetabulum and has remained the ‘gold standard’ ever since [12]. In the last two decades, many modifications in the surgical approaches to the anterior aspects of the acetabulum have been described [15–24]. The primary objective of the modified approaches was to reduce the surgical soft tissue damage and known complications. The optimization of the reduction quality has only emerged in recent times, as the ACPH fracture has become a common occurrence, as previously described. With appropriate surgical quality, the long-term results in terms of quality of life and mobility justify osteosynthesis, even in older patients. In order to fulfil the specific requirements of the fracture morphology in an ACPH pattern with regard to reduction quality, postoperative stability and low morbidity, the modified Stoppa and Pararectus approaches have been used more and more frequently, in addition to the ilioinguinal approach.

A surgical approach should enable the following surgical aims:

- Good accessibility of all fracture aspects.
- Best possible reduction quality.
- Optimal implant placement.
- High postoperative stability.
- Gentle surgical procedure.
- Low complication rate.

The Pararectus approach can fulfill these requirements in most cases of acetabular fracture involving the anterior column, but also in 2-column, T-type and ACPH cases. Moreover, a new generation of implants has been developed to address the specific requirements of older patients with acetabular fractures.

## Surgical technique

The Pararectus surgical technique, introduced by Keel et al. in 2012, serves as an alternative to the standard ilioinguinal approach and the modified Stoppa approach [19; 20; 25]. Two aspects of the technique were in of particular interest: (a) a single incision to reduce surgical trauma, (b) the optimal accessibility of the fracture and reduction quality.

**Fig. 2:**
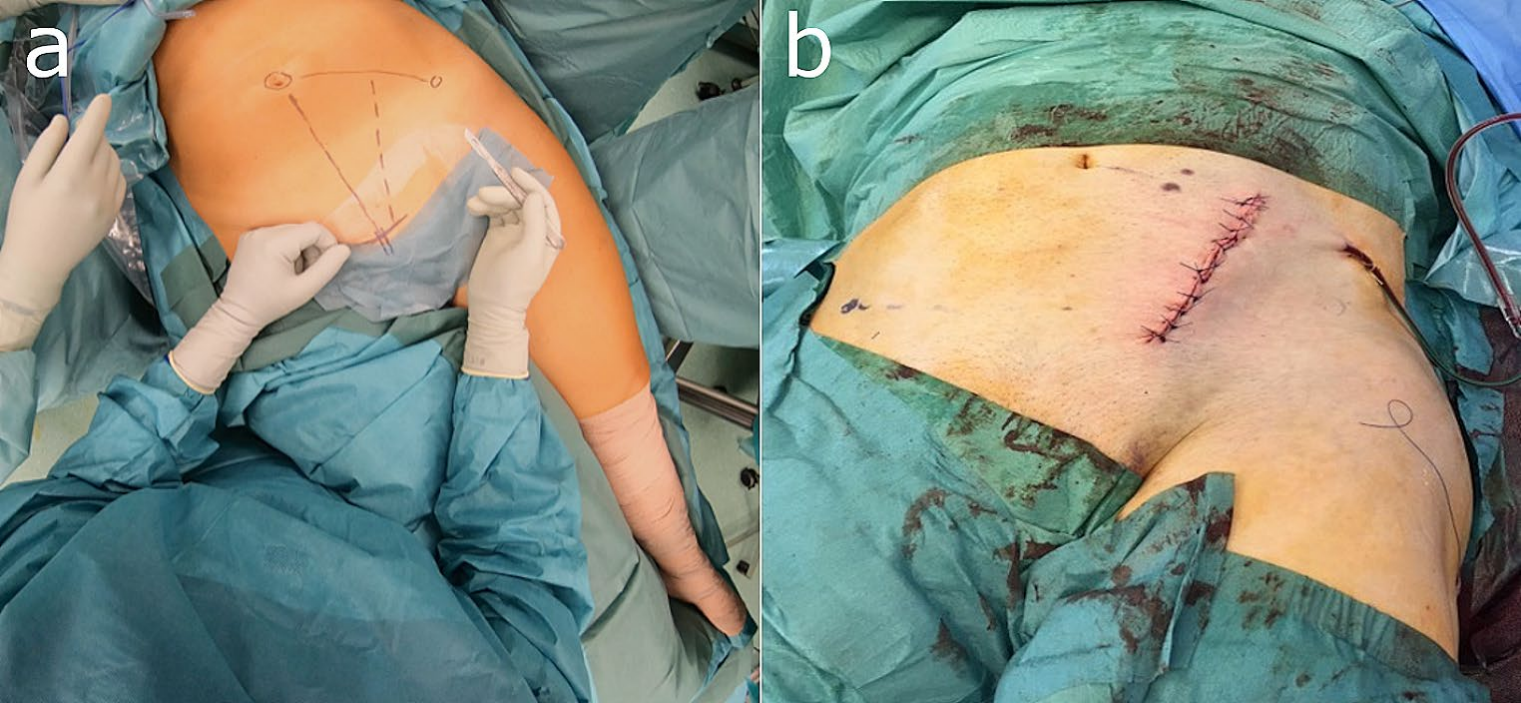
Pararectus approach: **a** Landmarks and **b** Maximum incision length

**Fig. 3:**
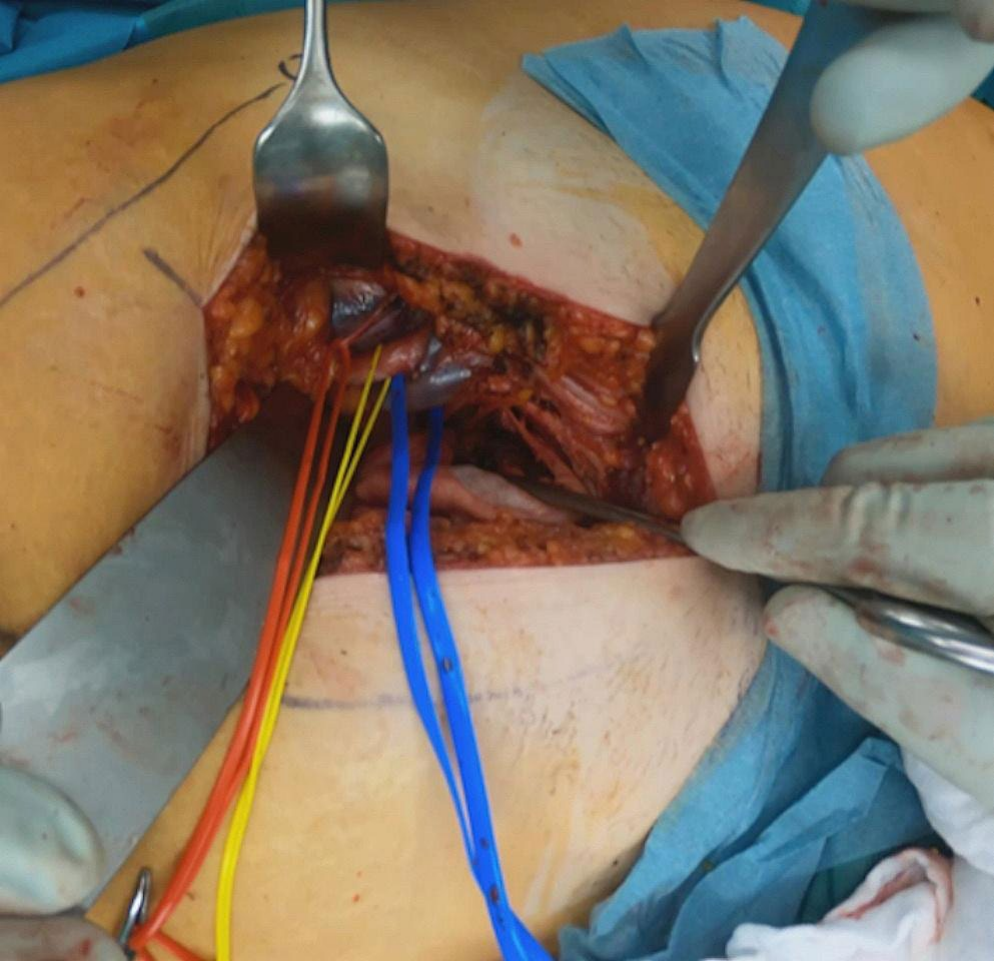
Pararectus approach: Intraoperative photograph demonstrating exposure and securing of the external iliac vessels with vessel loops

The surgical technique is as follows: a skin incision approximately 10 cm in length is made in the lateral third of an imaginary triangle between the umbilicus, the anterior superior iliac spine (ASIS) and the symphysis (Fig. 2a landmarks and Fig. 2b incision). Once the deep layer of the anterior abdominal wall has been reached, it is opened over the respective rectus abdominis muscle. The anterior rectus sheath is incised, and the transversalis fascia is opened, orienting on the lateral border of the rectus abdominis muscle. Subsequently, the extraperitoneal space is developed with meticulous blunt dissection. The vascular bundle, comprising the external iliac vein and artery, can now be isolated and secured in a safe manner accordingly using an elastic vessel loop. It is important to ensure that traction on the vessel loop is kept to a minimum throughout the operation, in particular to prevent thrombotic events (Fig. 3). The iliopsoas muscle is then dissected free from its surrounding tissues, while ensuring the protection of the local nerve structures. This can be accomplished by ensuring that the respective leg on the fracture side is flexibly covered during positioning. It is advisable to place a sterile, soft roll under the knee in order to reduce the tension on the hip flexors through flexion. The iliopectineal fascia is then incised and, as in the Stoppa approach, the superior ramus of the pubis is exposed. Particular attention must be paid to the vascular anastomosis of the epigastric or external iliac and obturator vessels, the so-called ‘corona mortis’. Its ligation is required in order to avoid bleeding and enable safe placement of the implants. The next step in the surgical procedure is the development of the “true pelvis” and thus, the exposure of the quadrilateral plate and further posterior advancement along the pelvic brim to the anterior aspects of the sacroiliac joint.

Once the exposure is complete, a large part of the acetabular roof can be surveyed by displacing the iliopsoas muscles laterally, thereby providing access to the displaced superomedial joint surface (medial wing of the gull sign). The latter can then be reduced directly via the fracture. In his surgical technique, Keel describes the development of a total of five windows, which can be achieved through the appropriate displacement of the respective structures and the use of suitable retractors [20]. A considerable portion of the iliac wing can be reached in the cranio-dorsal region by medializing or lateralizing the iliopsoas musculature. Caudal to the external iliac vessels, by displacing the vas deferens or ligamentum rotundum, the anterosuperior aspects of the anterior pelvic ring become accessible along the superior pubic ramus up to the symphysis. The quadrilateral plate can be visualized in depth and all essential fracture components can be reached via these five windows, particularly in ACPH fracture patterns [20].

### Accessibility of ACPH fracture components and quality of reduction

Despite the single incision, the Pararectus approach enables a comprehensive overview of the main components of the ACPH fracture pattern. Both the comminuted fracture in the area of the anterior column and the quadrilateral plate are directly accessible. The quadrilateral plate can be seen in the true pelvis down to the deep structures and can serve as a key fragment to indicate the quality of the reduction. The impacted superomedial dome fragment can be partially localized via the fracture from the medial side, although direct control of the fragment under vision is not possible. However, experience indicates that this approach allows for better control of the superomedial dome fragment than the ilioinguinal and modified Stoppa approaches. This is in line with the experience of Keel et al. [20]. Nevertheless, the data from the German Pelvic Registry did not show a clear advantage in reduction quality between the Pararectus and the Stoppa approach [26]. In the posterior aspect of the surgical field, the Pararectus approach allows for excellent visualization of the structures up to the SI joint, including the bifurcation of the internal iliac vessels and the lumbar plexus.

### Implant positioning and stability

In the original paper, Keel et al. utilized classic pelvic reconstruction plates [20]. In recent clinical practice and publications, the use of anatomical plates with support of the quadrilateral surface has been increasing. These can be inserted very precisely and safely via the Pararectus approach, while protecting the obturator nerve, vessels and large nerves. The first author’s experience suggests that employing the Pararectus approach with anatomical plates is a better option than using the Stoppa approach, as all relevant structures can be viewed directly. The anatomical flap systems within the Pararectus approach can also be used as a repositioning aid for the quadrilateral plate, in order to fit the medialized and rotated quadrilateral plate laterally. However, the Pararectus approach is disadvantageous for the exact placement of an antegrade posterior column screw. While the entry point is typically accessible even in obese patients, reaching the target corridor is hindered by the lateral soft tissues as a consequence of the more medial nature of the Pararectus approach. This task is considerably facilitated through the so-called 1st window of the other two approaches.

### Soft tissue trauma resulting from the surgical procedure

The expected soft tissue trauma resulting from the Pararectus approach is relatively minimal due to the short incision length (Fig. 2b) and the direct approach.

### Follow-up treatment

The overall condition of the patient is a prerequisite for the decision to perform ORIF, even in older patients. This also encompasses the patient’s mental and physical condition with regard to the implementation of the post-treatment regime. The patient must be mentally and physically capable of performing partial weight bearing of max. 20 kg with the assistance of walking aids over a period of 6 weeks. It is recommended that thrombosis prophylaxis with low-molecular-weight heparins or oral anticoagulants be administered for the period up to full weight-bearing is recommended, as is the administration of ossification prophylaxis. In addition, accompanying physiotherapy is recommended.

### Tips

Preoperative preparation:

1. Laxative measures and Simeticone administration: Administer mild laxatives to ensure bowel clearance and utilize sab simplex^®^ to minimize intestinal gas and improve visibility during imaging.
2. CT imaging: Perform a CT of the pelvis with femoral subtraction in the 3D reconstruction.
3. 3D printing: Consider 3D printing the fractured side of the pelvis for a detailed anatomical model, aiding in preoperative planning.
4. Anesthesia considerations: For patients unsuitable for general anesthesia with intubation, epidural anesthesia is a viable option due to the incision of the Pararectus approach.

Intraoperative techniques:

1. Positioning: Position the patient on a carbon fiber operating table to minimize imaging artifacts.
2. Surgical draping: Flexibly drape and position the surgical side or leg to maintain sterility while allow necessary moment during the procedure.
3. Urinary catheterization.
4. Intraoperative Imaging: Utilize intraoperative 3D scanning to provide real-time imaging, facilitating verification of anatomical reduction and correct implant placement.
5. Vessel management: Apply vessel loops around major vessels with minimal tension and only for short durations to reduce the risk of vascular injury and ensure adequate blood flow.

## Discussion

Over the past decade, we have observed a notable shift in the distribution of fracture types. The fracture types predominantly involving the anterior aspect of the acetabulum are significantly more common in patients over 60 years of age. The specific fracture morphology of the ACPH fracture has increasingly become the focus of attention due to its rising incidence and the specialized treatment it necessitates. Fixation of the quadrilateral surface is often described as difficult and is considered by some authors as one of the decisive prognostic factors for the long-term outcome, along with dislocation of the dome fragment. A 2013 systematic review published in the journal *Injury* analyzed all English-language publications addressing this particular anatomical region. Conservative treatment was compared with surgical treatment and the different surgical methods. A total of 1,573 studies were analyzed, of which 16 were included. The study analyzed publications between 1956 and 2012. The main finding of the study was that until the 1960s, conservative treatment was mainly propagated, but this required a long period of partial weight bearing and sometimes bed rest with extension [9]. However, as injuries to the quadrilateral surface increasingly affect older patients, the risks of concomitant problems such as thrombosis, cardiovascular failure or pneumonia are increased. Therefore, this treatment is no longer recommended [27; 28].

According to this analysis, surgical treatment is increasingly recommended in the more recent literature, but the fixation of the quadrilateral surface is often described as a major problem. Some authors consider the inadequate reduction to be a problem of the correct choice of approach. For others, the problem lies in the stable support of the quadrilateral plate [9]. A distinctive feature of the ACPH fracture is the frequently dislocated superomedial dome fragment, which plays a critical role in centering the femoral head. If the superomedial dome is missing, the femoral head follows the fracture and is cranialized and medialized by the traction of the muscles surrounding the hip. This constellation is very challenging for optimal surgical treatment because of the difficulty in accessing the fracture pathology, especially the superomedial dome fragment. Poor reduction quality and insufficient stabilization of this fragment often result in poor long-term patient outcomes. Assuming that the results of the German Pelvic Trauma Registry are representative of other countries with a longer life expectancy, the question arises as to which approach is best suited to achieve the goals of surgical treatment.

Taking the most common type of fracture, ACPH, as an example, the following main goals can be formulated that should be achieved by the chosen approach:

1. Restoration of the center of rotation of the hip joint.
2. Exact reconstruction of the articular surface without residual steps or gaps.
3. Exercise-stable osteosynthesis.

According to the analysis of the data from the German Pelvic Registry by Küper et al. [26], the first two main objectives can be achieved comparably well using both the modified Stoppa approach and the Pararectus approach. This is not consistent with the results of Keel et al., who were able to show a significant improvement in reduction quality via the Pararectus approach in their work [19; 20]. However, the quality of reduction achievable will be primarily dependent on the surgeon’s experience with the respective approach. The key to achieving the most accurate reduction in ACPH fractures lies in the precise fitting of the dome fragment during reduction. Due to the fracture mechanism, this superomedial acetabular roof fragment becomes cranialized, tilted and displaced from its stabilizing connections to the pelvic brim and quadrilateral plate. Proper relocation of the femoral head hinges on the exact reduction of this fragment, which involves caudalization and correction of the tilt. The chosen surgical approach must facilitate these maneuvers. The modified Stoppa approach only allows this via the additional so-called 1st window of the ilioinguinal approach. In comparison, this maneuver is usually directly possible, when using the Pararectus approach. The second critical step is the permanent stabilization of the dome fragment. This is accomplished by ‘slamming’ the ventrally ‘door-like’ opened quadrilateral surface. The transition between the pelvic brim and the quadrilateral plate represents the load-bearing beam. This also stabilizes the dome fragment, if it is accurately restored. The quadrilateral plate itself does not serve a load-bearing function; rather, it serves as the contact surface for the reduction. Both the modified Stoppa and the Pararectus approaches are well-suited for this procedure, as both can be employed to reach the medial surface of the quadrilateral plate. It can be argued that the ilioinguinal approach is inferior, as there is less direct access to the quadrilateral plate.

Another main objective of optimized treatment is long-term stabilization of the fracture components. In a study by Laflamme et al. [29], anatomical reduction was achieved in 52.4% of patients using a modified Stoppa approach. Nevertheless, in 4 cases, there was a secondary loss of reduction, resulting in a rapid progression of osteoarthritis. Chen et al. [30] and Farid [31] describe the use of a wire or cable cerclage for additional protection and improved support.

From this development, the so-called spring plate emerged as an effective instrument for preventing medial loss of reduction. This was subsequently superseded by plate models that were adapted to the anatomy, as well as plate systems that have a configuration that allows for the support of the quadrilateral surface. These have become widespread since their introduction. These plate systems can be placed very well using all the aforementioned approaches. It should be noted that there are differences between the individual approaches with regard to screw placement, particularly of the screws close to the SI-joint in the cranio-dorsal parts of the plates. Using the Stoppa approach, orthograde placement of these screws is only possible via the additional access of the 1st window. By using the Pararectus approach, this can be achieved via the single approach, with limitations only arising in significantly overweight patients.

The ilioinguinal approach is associated with a high rate of procedure-related complications, including soft tissue injury, injury to local nerves, and the risk of an inguinal hernia [16; 32]. A comparison of the access-associated comorbidities reveals some differences between the approaches. It is evident that the extent of soft tissue damage is contingent upon the approach employed. The incision for the ilioinguinal approach is typically between 30 and 40 cm in length. When the incision of the modified Stoppa approach is combined with its frequently required extension (1st window), the incision adds up to a total length of approximately 20 cm. In contrast, the incision of the Pararectus approach is only ranging between 8 and 12 cm.

The literature indicates that both limited approaches (Stoppa/ Pararectus) are associated with less access-related nerve damage, particularly that of the lateral cutaneous nerve [20; 26]. However, the significance between the Stoppa and Pararectus has not been clearly elucidated yet [26] and is rather strongly dependent on intraoperative handling for both accesses. The incidence of postoperative hernia formation varies considerably depending on the approach employed and must be considered in a nuanced manner.

The ilioinguinal approach involves a direct route in the area of the inguinal ligament and the inguinal canal, which may potentially lead to the formation of an inguinal hernia postoperatively [32]. The formation of hernias as a consequence of the Stoppa and Pararectus have not yet been described in the medical literature. Nevertheless, there are so-called abdominal wall relaxations that have a neurogenic etiology. These can occur particularly in the lateral abdominal wall, in the ilioinguinal area, as well as in the first window of the Stoppa approach and are very difficult to treat. The occurrence of diastases of neurogenic origin in the Pararectus approach has not yet been described.

The decision regarding the most suitable approach for patients with previously treated inguinal hernias must be made on a case-by-case basis, with the most appropriate approach being determined by the specific hernia repair procedure. In classical procedures such as those described by Shouldice or Lichtenstein, the Stoppa and Pararectus approaches are preferable to the ilioinguinal approach. This is due to the anatomical relationship to the inguinal canal, as also described by Keel [20]. All three approaches are more challenging in the context of contemporary surgical techniques, which employ the so-called transperitoneal (TAPP) and extraperitoneal (TEPP) approaches with the implantation of a mesh. The anatomical structures are also very difficult to visualize with Pararectus approach due to the size of the mesh inserted and its attachment to the upper pubic ramus. This will present an additional challenge in anterior acetabular surgery in the future, as the trend and the study situation in visceral surgery are clearly in favor of these treatment techniques.

## Conclusion

In recent years, two significant observations have been made in the treatment of acetabular fractures. Firstly, there has been a notable increase in the proportion of acetabular fractures involving the anterior column. This rise is possibly due to demographic shifts and the increased mobility of the golden ager. Secondly, there have been advancements in surgical techniques, including the development of less invasive approaches, new implants and improvements in perioperative management.

Recent studies, indicate a trend to less invasive approaches. However, interestingly, none of these studies have demonstrated a significant reduction in morbidity across all patients. This may be attributed to the learning curve and the time required for surgeons to adapt to new procedures. Additionally, it is important to recognize that the average age of patients undergoing treatment for acetabular fractures is steadily increasing. Given the increasing age and associated fragility of these patients, it is crucial to consider all aspects when deciding on the appropriate treatment option. The fragility of the physiological steady state in elderly patients necessitates a careful and comprehensive evaluation to ensure the chosen treatment maximizes outcomes while minimizing risks.

## References

[1] Judet R, Judet J, Letournel E (1964) Fractures of the Acetabulum: classification and Surgical approaches for Open Reduction. Preliminary Report. J Bone Joint Surg Am 46:1615–1646 14239854

[2] Judet R, Judet J, Letournel E (1964) [Fractures of the Acetabulum]. Acta Orthop Belg 30:285–293 14201066

[3] Tscherne H, Regel G, Pape HC, Pohlemann T, Krettek C (1998) Internal fixation of multiple fractures in patients with polytrauma. Clin Orthop Relat Res (347):62–78 9520876

[4] Blum J, Beyermann K, Ritter G (1991) [Incidence of acetabular fractures before and after introduction of compulsory seatbelt fastening]. Unfallchirurgie 17(5):274–279 1962371 10.1007/BF02588406

[5] Letournel E (1980) Acetabulum fractures: classification and management. Clin Orthop Relat Res (151):81–106 7418327

[6] Letournel E (1993) The treatment of acetabular fractures through the ilioinguinal approach. Clin Orthop Relat Res (292):62–76 8519138

[7] Ochs BG, Marintschev I, Hoyer H et al (2010) Changes in the treatment of acetabular fractures over 15 years: analysis of 1266 cases treated by the German Pelvic Multicentre Study Group (DAO/DGU). Injury 41(8):839–851 20451195 10.1016/j.injury.2010.04.010

[8] Pohlemann T, Stengel D, Tosounidis G et al (2011) Survival trends and predictors of mortality in severe pelvic trauma: estimates from the German pelvic trauma Registry Initiative. Injury 42(10):997–1002 21513936 10.1016/j.injury.2011.03.053

[9] White G, Kanakaris NK, Faour O, Valverde JA, Martin MA, Giannoudis PV (2013) Quadrilateral plate fractures of the acetabulum: an update. Injury 44(2):159–167 23121991 10.1016/j.injury.2012.10.010

[10] Rommens PM, Wagner D, Hofmann A (2012) [Osteoporotic fractures of the pelvic ring]. Z fur Orthopadie Und Unfallchirurgie 150(3):e107–118 quiz e119-120 10.1055/s-0032-1314948 22723074

[11] Rommens PM, Wagner D, Hofmann A (2012) Surgical management of osteoporotic pelvic fractures: a new challenge. Eur J Trauma Emerg Surgery: Official Publication Eur Trauma Soc 38(5):499–509 10.1007/s00068-012-0224-8 PMC3495273 23162670

[12] Matta JM (1994) Operative treatment of acetabular fractures through the ilioinguinal approach. A 10-year perspective. Clin Orthop Relat Res (305):10–19 8050218

[13] Hessmann MH, Nijs S, Rommens PM (2002) [Acetabular fractures in the elderly. Results of a sophisticated treatment concept]. Unfallchirurg 105(10):893–900 12376896 10.1007/s00113-002-0437-0

[14] Ferguson TA, Patel R, Bhandari M, Matta JM (2010) Fractures of the acetabulum in patients aged 60 years and older: an epidemiological and radiological study. J Bone Joint Surg Br 92(2):250–257 20130318 10.1302/0301-620X.92B2.22488

[15] Cole JD, Bolhofner BR (1994) Acetabular fracture fixation via a modified Stoppa limited intrapelvic approach. Description of operative technique and preliminary treatment results. Clin Orthop Relat Res (305):112–123 8050220

[16] Helfet DL, Schmeling GJ (1994) Management of complex acetabular fractures through single nonextensile exposures. Clin Orthop Relat Res (305):58–68 8050248

[17] Hirvensalo E, Lindahl J, Kiljunen V (2007) Modified and new approaches for pelvic and acetabular surgery. Injury 38(4):431–441 17445529 10.1016/j.injury.2007.01.020

[18] Jakob M, Droeser R, Zobrist R, Messmer P, Regazzoni P (2006) A less invasive anterior intrapelvic approach for the treatment of acetabular fractures and pelvic ring injuries. J Trauma 60(6):1364–1370 16766988 10.1097/01.ta.0000208139.97474.f7

[19] Keel MJ, Bastian JD, Buchler L, Siebenrock KA (2013) [Anterior approaches to the acetabulum]. Unfallchirurg 116(3):213–220 23478898 10.1007/s00113-012-2332-7

[20] Keel MJ, Ecker TM, Cullmann JL et al (2012) The Pararectus approach for anterior intrapelvic management of acetabular fractures: an anatomical study and clinical evaluation. J Bone Joint Surg Br 94(3):405–411 22371551 10.1302/0301-620X.94B3.27801

[21] Kloen P, Siebenrock KA, Ganz R (2002) Modification of the ilioinguinal approach. J Orthop Trauma 16(8):586–593 12352568 10.1097/00005131-200209000-00008

[22] Ponsen KJ, Joosse P, Schigt A, Goslings JC, Luitse JS (2006) Internal fracture fixation using the Stoppa approach in pelvic ring and acetabular fractures: technical aspects and operative results. J Trauma 61(3):662–667 16967004 10.1097/01.ta.0000219693.95873.24

[23] Sagi HC, Afsari A, Dziadosz D (2010) The anterior intra-pelvic (modified rives-stoppa) approach for fixation of acetabular fractures. J Orthop Trauma 24(5):263–270 20418730 10.1097/BOT.0b013e3181dd0b84

[24] Tannast M, Siebenrock KA (2009) [Operative treatment of T-type fractures of the acetabulum via surgical hip dislocation or Stoppa approach]. Oper Orthop Traumatol 21(3):251–269 19779682 10.1007/s00064-009-1803-7

[25] Keel MJB, Siebenrock KA, Tannast M, Bastian JD (2018) The Pararectus Approach: a New Concept. JBJS Essent Surg Tech 8(3):e21 30588366 10.2106/JBJS.ST.17.00060 PMC6292723

[26] Küper MA, Röhm B, Audretsch C et al (2022) Pararectus approach vs. Stoppa approach for the treatment of acetabular fractures - a comparison of approach-related complications and operative outcome parameters from the German Pelvic Registry. Orthop Traumatol Surg Res 108(4):103275 35331921 10.1016/j.otsr.2022.103275

[27] Toro JB, Hierholzer C, Helfet DL (2004) Acetabular fractures in the elderly. Bull Hosp Jt Dis 62(1–2):53–57 15517858

[28] Cornell CN (2005) Management of acetabular fractures in the elderly patient. HSS J 1(1):25–30 18751805 10.1007/s11420-005-0101-7 PMC2504130

[29] Laflamme GY, Hebert-Davies J, Rouleau D, Benoit B, Leduc S (2011) Internal fixation of osteopenic acetabular fractures involving the quadrilateral plate. Injury 42(10):1130–1134 21156315 10.1016/j.injury.2010.11.060

[30] Chen CM, Chiu FY, Lo WH, Chung TY (2001) Cerclage wiring in displaced both-column fractures of the acetabulum. Injury 32(5):391–394 11382424 10.1016/s0020-1383(00)00243-6

[31] Farid YR (2010) Cerclage wire-plate composite for fixation of quadrilateral plate fractures of the acetabulum: a checkrein and pulley technique. J Orthop Trauma 24(5):323–328 20418739 10.1097/BOT.0b013e3181c90bbe

[32] Rommens PM, Hessmann MH (1999) [Acetabulum fractures]. Unfallchirurg 102(8):591–610 10484903 10.1007/s001130050455

